# IRE1α pathway: A potential bone metabolism mediator

**DOI:** 10.1111/cpr.13654

**Published:** 2024-05-12

**Authors:** Chengbo Yu, Zhixiang Zhang, Li Xiao, Mi Ai, Ying Qing, Zhixing Zhang, Lianyi Xu, Ollie Yiru Yu, Yingguang Cao, Yong Liu, Ke Song

**Affiliations:** ^1^ Department of Stomatology, Tongji Hospital, Tongji Medical College Huazhong University of Science and Technology Wuhan China; ^2^ Department of Prosthodontics and Implantology, School of Stomatology, Tongji Medical College Huazhong University of Science and Technology Wuhan China; ^3^ Hubei Province Key Laboratory of Oral and Maxillofacial Development and Regeneration Wuhan China; ^4^ Faculty of Dentistry The University of Hong Kong Hong Kong SAR China; ^5^ Hubei Key Laboratory of Cell Homeostasis, College of Life Sciences, Frontier Science Center for Immunology and Metabolism, and the Institute for Advanced Studies Wuhan University Wuhan Hubei China

## Abstract

Osteoblasts and osteoclasts collaborate in bone metabolism, facilitating bone development, maintaining normal bone density and strength, and aiding in the repair of pathological damage. Endoplasmic reticulum stress (ERS) can disrupt the intracellular equilibrium between osteoclast and osteoblast, resulting in dysfunctional bone metabolism. The inositol‐requiring enzyme‐1α (IRE1α) pathway—the most conservative unfolded protein response pathway activated by ERS—is crucial in regulating cell metabolism. This involvement encompasses functions such as inflammation, autophagy, and apoptosis. Many studies have highlighted the potential roles of the IRE1α pathway in osteoblasts, chondrocytes, and osteoclasts and its implication in certain bone‐related diseases. These findings suggest that it may serve as a mediator for bone metabolism. However, relevant reviews on the role of the IRE1α pathway in bone metabolism remain unavailable. Therefore, this review aims to explore recent research that elucidated the intricate roles of the IRE1α pathway in bone metabolism, specifically in osteogenesis, chondrogenesis, osteoclastogenesis, and osteo‐immunology. The findings may provide novel insights into regulating bone metabolism and treating bone‐related diseases.

## Graphical abstract

Numerous prospective avenues exist for research on the IRE1α pathway. The IRE1α pathway can regulate osteogenesis, chondrogenesis, and osteoclatogenesis. IREI α is implicated in development of osteoblast, osteoclast, and chondrocyte and bone‐immune interaction.

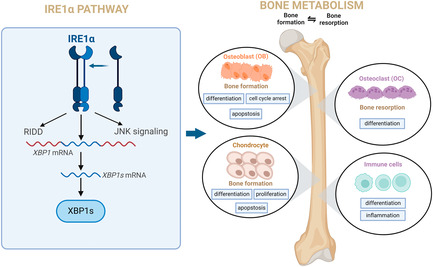

## 1 INTRODUCTION

The skeletal system stands as one of the most fundamental components of the human body. Beyond supporting and defending the human body, it plays vital physiological functions, including haematopoiesis and storage of essential minerals.^1^, ^2^ Bone metabolism is characterized by a finely tuned collaboration between bone cells—osteoblasts, osteoclasts, osteocytes, and chondrocytes—working in concert to maintain bone tissue quantity and the structural integrity of bones.^3^ This process represents a dynamic equilibrium. Osteoblasts synthesize and secrete collagen and minerals, while osteoclasts function by degrading bone matrix via acids and enzyme secretion. Chondrocytes are the only cell type within mature cartilage tissue, playing pivotal roles in cartilage metabolism. These cells (osteoblasts, chondrocytes, and osteoclast) interact with each other through producing cytokines^4^ and receiving exosomes derived from other bone cells to restore bone homeostasis.^5^ In recent years, many reports have shown that immune cells in the bone immune microenvironment are also involved in the regulation of bone metabolism.^6^ An imbalance between bone production and resorption leads to conditions such as osteoporosis and sclerosteosis. Numerous signalling pathways exert regulatory effects on bone metabolism and the three bone cell types mentioned above. The pathways include wingless‐type MMTV integration site family,^7^ hedgehog (Hh),^8^ notch,^9^ transforming growth factor‐β (TGF‐β),^10^ adenosine 5′‐monophosphate (AMP)‐activated protein kinase (AMPK)^11^ and so on. Our previous research indicates that Hh promotes bone formation^12^, ^13^, ^14^ and interacts with TGF‐β.^15^

Furthermore, although significant progress has been made such as bone regeneration, there are still many obstacles in the way of treatment of bone metabolism disorders.^16^ New therapeutic methods for bone regeneration emerge in an endless manner, among which bioactive elements and materials^17^, ^18^, ^19^, ^20^ are particularly prominent. In vivo, osteo‐organoids constructed by BMP‐2‐loaded gelatin sponge scaffolds could even serve as a stable source of mesenchymal stem cells (MSCs) with large quantity, high purity, and strong stemness.^21^ The combination of the clinical application of pathways as therapeutic targets and the technology of bioactive materials may yield an excellent efficacy of one plus one greater than two. Therefore, it is also believed that understanding molecular signalling pathways involved in bone metabolism is crucial to the development of these novel therapeutic technique in treating bone‐related diseases.^22^

The endoplasmic reticulum (ER), a vital cellular organelle responsible for protein processing, operates within a limited capacity. Cellular exposure to biological stressors such as hypoxia, low glucose levels, intracellular calcium fluctuation, and excessive protein synthesis can lead to ER damage. This damage results in an accumulation of unfolded proteins within the ER lumen. Osteoblasts, osteoclasts, and chondrocytes are involved in various protein synthesis and secretion to maintain bone homeostasis.^23^ Consequently, these cells constantly handle the challenge of managing the load from protein synthesis. Over time, excessive accumulation of unfolded or misfolded proteins triggers ER stress and unfolded protein response (UPR) activation to cope with this stress. In mammalian cells, the UPR is mediated by three main signalling branches: inositol‐requiring enzyme 1α (IRE1α), PKR‐like ER kinase (PERK), and activating transcription factor 6 (ATF6). Many studies have revealed the involvement of endoplasmic reticulum stress (ERS) and UPR pathways in osteogenesis and osteoclastogenesis. These pathways are pivotal in bone‐related and periodontal diseases.^24^, ^25^, ^26^, ^27^, ^28^

A surge in research on IRE1α signalling in bone metabolism has been witnessed in recent years. These studies further reveal the intricate molecular mechanisms involving the three cell types participating in bone metabolism, fostering further avenues for exploration in this field. The roles of the IRE1α pathway have been elucidated in osteogenesis, chondrogenesis, and osteclastogenesis.^29^, ^30^, ^31^ Furthermore, the osteo‐immune role of IRE1α pathway has also been unveiled.^32^, ^33^, ^34^ However, relevant reviews on these subjects remain unavailable. Therefore, this review aims to summarize the role of the IRE1α pathway in bone metabolism and its association with certain bone‐related diseases.

## 2 IRE1α PATHWAY

IRE1α pathway is considered the most conservative UPR pathway. IRE1α serves as a vital transmembrane ER stress sensor, The structure of the IRE1α can be divided into the following components: an N‐terminal luminal domain, a single‐pass transmembrane spanning segment, and a cytosolic region subdivided into a Ser/Thr protein kinase domain and C‐terminal endoribonuclease (RNase) domain.^35^, ^36^ Upon sensing ER stress, IRE1α dissociates from Binding immunoglobulin protein (BiP) and releases its C‐terminal domain.^37^ This process triggers the dimerization and autophosphorylation of IRE1α, thus activating its endonuclease activity.^38^, ^39^ Upon activation, IRE1α targets and facilitates the unconventional splicing of downstream substrate X‐box binding protein 1 (XBP1) mRNA. This process generates a spliced form of Xbp1 mRNA, which encodes a more stable and potent XBP1 protein.^40^, ^41^ The transcription factor XBP1 plays a crucial role in restoring ER homeostasis by promoting ER‐associated degradation (ERAD) of misfolded proteins and ER biogenesis.^36^, ^42^, ^43^ Degradation of several mRNAs and some pre‐miRNAs can also be facilitated by activated IRE1α RNase, which is a process known as regulated IRE1‐dependent decay (RIDD).^44^ Moreover, phosphorylated IRE1α can activate c‐Jun N‐terminal kinase (JNK) via its kinase activity,^45^ contributing to processes such as autophagy, apoptosis, and inflammation.^46^, ^47^

Over the past decade or two, the IRE1α pathway has emerged as a significant player in metabolic homeostasis and associated diseases, spanning conditions including obesity, hyperglycemia, and hyperlipidemia.^48^, ^49^, ^50^ This pathway also regulates lipid metabolism and glycometabolism across various organs.^49^, ^51^, ^52^, ^53^ We have explored the important metabolic effect of the IRE1α pathway in the liver,^51^, ^53^ adipose tissue,^48^, ^54^ and skeletal muscle.^55^ Besides, the involvement of IRE1α in bone formation, bone loss, and various bone diseases has been gradually unveiled.^56^, ^57^, ^58^ Therefore, exploring the potential regulatory role of the IRE1α pathway in bone metabolism and homeostasis could be crucial.

## 3 OSTEOGENESIS

### 3.1 Role of the IRE1α pathway in osteogenic differentiation

The utilization of recombinant bone morphogenetic protein 2 (BMP2) is prevalent in promoting osteogenic differentiation, a process that involves complex mechanisms.^59^ Under BMP2 induction, activated Smad (Smad1/4, Smad5/4) can be mobilized to the promoter region of XBP1, enhancing the transcriptional activity of its reporter gene. This suggests a potential mechanism underlying the activation of the IRE1α pathway during osteogenic differentiation.^60^ As per a recent study, IRE1α and its downstream transcription factor XBP1s play an essential role in BMP2‐induced osteoblast differentiation.^29^ When exposed to BMP2 induction, *Ern1* (the gene encoding IRE1α)−/− mouse embryonic fibroblasts (MEFs), where IRE1α is knocked out, exhibited substantial osteogenic differentiation defects than the control group. This is evident in the marked downregulation of osteogenic markers such as Osterix (Osx).^61^ The commitment of MSCs towards the chondro‐osteogenic phenotype relies on Osx and Runt‐related transcription factors (RUNX2).^62^ Specifically, RUNX2 has been demonstrated to act as an Osx regulator.^63^ The established interaction between the IRE1α pathway and RUNX2 has been substantiated,^64^, ^65^ suggesting a potential mechanism by which it could promote Osx expression by activating RUNX2. Furthermore, Tohmonda et al. identified Osx as an XBP1 target gene.^29^ Their luciferase and ChIP analysis revealed the binding of Xbp1 to the promoter region of the *Sp7* gene (which encodes Osx), subsequently enhancing Osx transcription. These findings collectively suggest that the IRE1α/XBP1‐dependent UPR pathway can facilitate osteogenesis by promoting Osx expression during BMP2‐induced osteogenesis. Moreover, a recent study on advanced glycation end products (AGEs) further proved the regulatory effect of IRE1α‐XBP1s on Osx.^66^ Another study revealed that XBP1s could also promote mineralization in ankylosing spondylitis (AS) MSCs, a type of axial inflammation.^67^ Cui et al. found that XBP1s promote the proliferation and osteogenesis of human periodontal ligament cells, an ideal group for periodontal tissue regeneration, by regulating autophagy and apoptosis.^68^

However, Guo et al. identified that IRE1α overexpression inhibits BMP2‐induced osteoblast differentiation in multipotent mesenchymal C2C12 cells while employing a siRNA approach to knockdown IRE1α enhances this process.^56^ Granulin epithelin precursor (GEP)^69^ and JunB have been identified as upstream activators of IRE1α during BMP2‐induced osteoblast differentiation, which establishes a complicated negative regulatory loop. BMP‐2 stimulates C2C12 cells and primary BMSCs to activate GEP‐JunB‐IRE1α axis, while IRE1α, in turn, suppresses BMP‐2 and GEP‐induced osteoblastogenesis.^56^ Zhang et al. also suggested that the IRE1α‐mediated UPR may alleviate the ossification of MEFs by attenuating Shh signalling.^70^ Sonic hedgehog N‐terminus (N‐Shh) is an agonist of Shh pathway. Treatment of N‐Shh facilitated osteogenic activity (ALP activity, matrix mineralization, and the expression of Alp and Col‐I) in MEFs under osteogenic induction, during which IRE1α expression was inhibited. Conversely, deletion of IRE1α leads to further activation of Shh pathway and promotes the osteogenic effect of N‐Shh,^70^ indicating inhibitory effect of IRE1α on osteogenesis may be achieved through mediating Shh signalling.

In recent years, the proteasome inhibitor (PI) has emerged as one of the most effective medications for treating multiple myeloma (MM). This significantly improves the overall survival rate of patients with MM. The targeted PI is believed to maintain bone metabolism by inhibiting bone resorption while promoting bone formation.^71^, ^72^, ^73^ Recently, its mechanism has been associated with the IRE1α pathway. Bortezomib—an effective PI utilized in certain tumours—was observed to significantly increase Xbp1 splicing across four cell types analysed: mouse bone marrow MSCs, MSCs derived from patients with MM (MM‐MSCs), MC3T3‐E1, and human MSC line immortalized by the telomerase reverse transcriptase gene expression.^74^, ^75^ Inhibition of IRE1αimpedes the PI‐induced osteogenic differentiation.^74^ Conversely, bortezomib has been demonstrated to promote femur bone formation by activating the XBP1s pathway in 5‐week‐old mice.^74^ The IRE1α‐XBP1 pathway clearly mediates the bone formation effect of bortezomib. However, how XBP1s stimulate osteogenesis in the presence of bortezomib remains unclear.

A recent study might partially address this question. Cell cycle withdrawal has been incorporated into PI‐induced osteogenic differentiation, a process related to the IRE1α pathway.^76^ Bortezomib was found to induce G0/G1 phase cell cycle arrest and significantly upregulated p21^Cip1^ and p27^Kip1^ expression. (negative regulators of progression from G1 to S phase)^77^, ^78^ at the protein and mRNA levels during osteogenesis. Bortezomib was shown to activate XBP1s, leading to upregulation of p21^Cip1^ and p27^Kip1^ expression via binding to their promoters. This process resulted in cell cycle arrest, facilitating the subsequent entry into osteogenic differentiation.^76^ Targeting cell cycle withdrawal appears promising for investigating osteogenic differentiation.

These findings collectively suggest that the IRE1α‐XBP1 pathway has at least two roles in osteoblast differentiation. First, it aids in managing the protein‐processing load in the endoplasmic reticulum. Second, it directly regulates BMP2‐induced osteogenic differentiation. However, considering the opposing roles played by IRE1α in distinct cell models, determining whether it acts as a positive or negative regulator might depend on cell type and complexity of the in vivo microenvironment. Last but not least, a new role for IRE1α in osteogenesis beyond the previously mentioned roles has been proposed: regulating cell cycle withdrawal. Above all, further research is necessary to determine whether and how the IRE1α pathway regulates this process during osteogenesis under physiological or pathological conditions. A recent investigation^79^ indicated that exosome carrying Wnt agonist 1 could alleviate bone loss and promote bone formation in colitis mice, using exosome loaded with IRE1α agonist or antagonist to treat bone‐related disease may be beneficial to identify the accurate effect of IRE1α pathway on in vivo osteogenesis.

### 3.2 Involvement of the IRE1α pathway in osteoblast apoptosis

In addition to osteoblast proliferation and differentiation, osteoblast apoptosis plays a crucial role in bone metabolism. Prolonged or severe dysfunction in ER ultimately leads to cell death.^80^ The ER is associated with three recognized proapoptotic pathways, mediated by IRE1α, caspase‐12, and PERK/C/EBP‐homologous protein (CHOP), respectively. During IRE1α‐mediated apoptosis, the activated IRE1α interacts with JNK, subsequently recruiting Tumour necrosis factor Receptor Associated Factor 2 (TRAF2) and Apoptosis signal‐regulating kinase 1 (ASK1) to the ER membrane to form an apoptotic complex. Subsequently, the downstream target JNK is activated as an important pro‐apoptotic signal.^81^ Modulating IRE1α signalling ameliorates osteoporotic osteoblast apoptosis through an IRE1‐ASK1‐JNK axis.^82^ Additionally, IRE1‐dependent decay of mRNA may also contribute to IRE1α‐mediated cell apoptosis.^83^, ^84^ ER stress is implicated in osteoblast migration in diseases exhibiting an osteogenic down‐regulation phenotype, including hyperhomocysteinemia, skeletal fluorosis, inflammation, and diabetes.^85^, ^86^, ^87^, ^88^, ^89^, ^90^ Furthermore, the IRE1α pathway was found to play a role in the cell death process associated with these diseases.

Homocysteine (Hcy) is a sulphur‐containing amino acid that plays a role in various biological processes. Hyperhomocysteinemia is considered to induce osteoporosis, with Hcy‐induced osteoblasts apoptosis responsible for the decline in bone formation.^91^ Based on research findings, Hcy can alleviate IRE1α expression, which is involved in Hcy‐induced cell death.^85^ JNK activity was not detected in this context. Conversely, reports indicate that Hcy‐induced IRE1α activation triggers rapid and sustained activation of JNK in endothelial cells.^92^, ^93^ Given that continuous JNK activation is associated with apoptosis, it is speculated that the protective effect of silencing IRE1α is partially due to the disruption of JNK activation. Melittin (MEL), a crucial component in the extract of bee venom (BV), a non‐steroidal anti‐inflammatory drug, possesses the ability to trigger apoptosis.^94^, ^95^ Studies indicate that the IRE1α/XBP1 pathway is involved in MEL‐induced apoptosis in the osteoblast cell line MG63.^96^ MEL expression exclusively activated IRE1α in MG63 cells rather than PERK or ATF6, suggesting the involvement of an IRE1α‐XBP1‐CHOP pathway in apoptosis and growth suppression in MG63 cells. The non‐enzymatic interaction between glucose, ketose, or carbonyl chemicals with protein results in the formation of AGEs. These AGESs are recognized to accumulate in the bone matrix as people age. High‐dose extracellular AGEs can induce human fetal osteoblastic cell line (hFOB 1.19) apoptosis via the AGE‐The receptor for advanced glycation end products (RAGE) signalling pathway, negatively affecting bone production.^97^ Recently, IRE1α was found to be involved in AGEs‐induced osteoblast apoptosis.^66^, ^90^ AGEs have been observed to increase the phosphorylation of IRE1α, JNK, and p38 in MC3T3‐E1. However, IRE1α knockdown prevented the AGEs‐induced downregulation of the Bcl2/Bax ratio and caspase‐3 activation. Moreover, it improved cell viability compared to the control siRNA‐treated group.^90^ These findings suggest that AGEs trigger osteoblast death in an IRE1α‐dependent manner.

**FIGURE 1:**
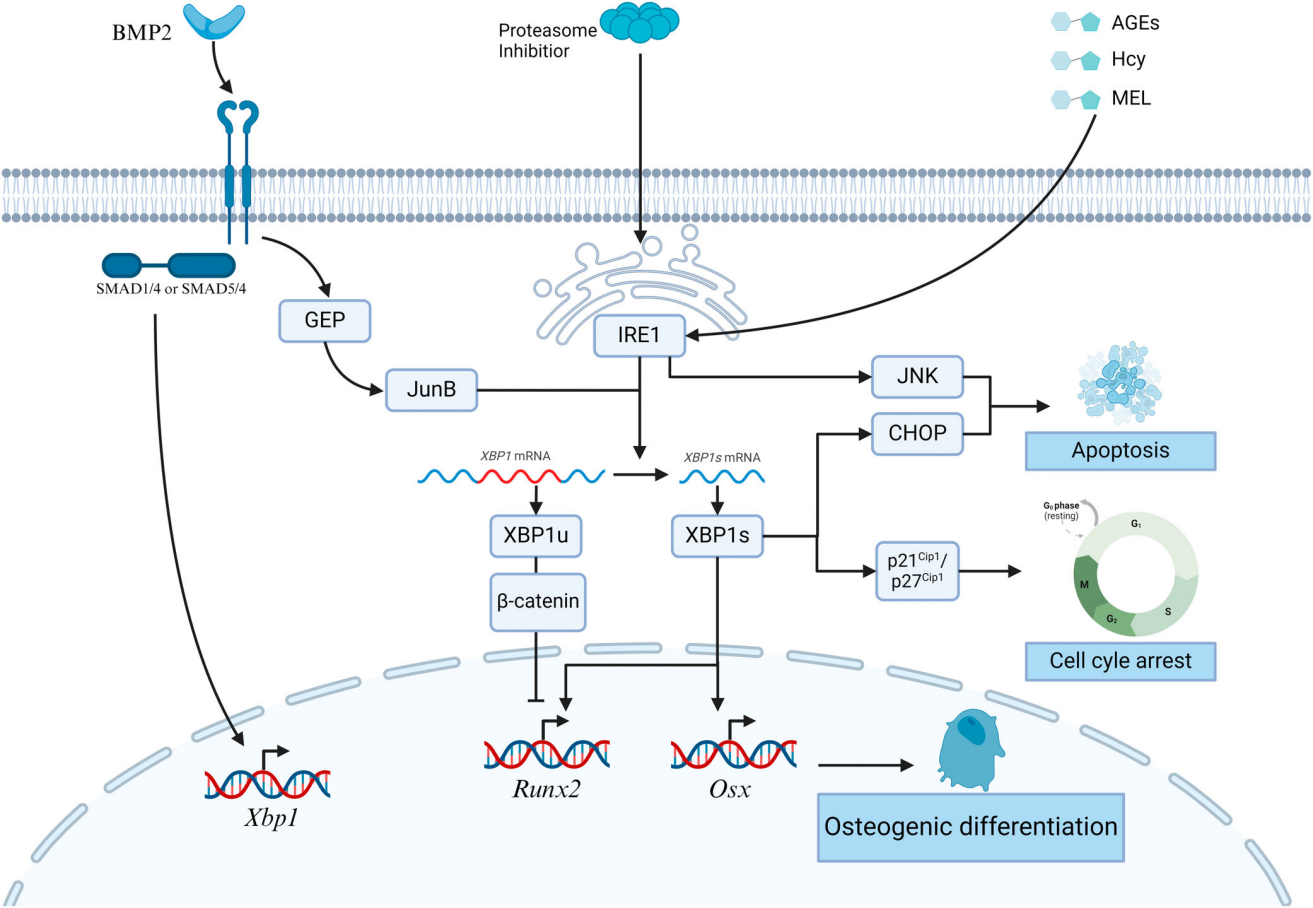
IRE1α pathway regulation in osteogenesis. XBP1u and XBP1s exhibit distinct effects on osteogenic differentiation, respectively. Downstream factors, including JNK and CHOP, are also involved in osteoblast apoptosis.

Given the involvement of the IRE1α pathway in osteoblast apoptosis during these pathological conditions, it becomes a potential therapeutic target for treating diabetes and osteoporosis. It also aids in reducing fracture risk. Thus, osteogenesis may be regulated by the IRE1α pathway (Figure 1). Some of the studies involving the IRE1α pathway and osteogenesis have also been summarized in Table 1.

**TABLE 1:** Role of IRE1α pathway in osteogenesis.

Cell type/animal model	Results	Conclusion	Reference
MEFs, MC3T3‐E1	XBP1 was upregulated in BMP2‐induced osteoblast differentiation BMP2‐induced osteoblast differentiation is hampered by the absence of IRE1α XBP1 can bind to Osx and thapsigargin‐induced ER stress promoted Osx expression in an XBP1s‐dependent manner	IRE1α‐XBP1 pathway stimulate Osx expression to promote the maturation of preosteoblasts into osteoblasts	^30^
Wild type mouse	IRE1α was activated in the BMP2‐treated calvarial bone		^30^
ST2 cells	IRE1α was activated by TG to promote osteoblast differentiation and inhibited by AGEs	AGEs inhibited the differentiation of stromal cells into osteoblasts by suppressing ER stress sensors such as IRE1a, ATF6, and OASIS	^68^
C2C12 cells, BMSCs	Overexpression of IRE1a inhibits the BMP2‐mediated osteogenic differentiation Knockdown of IRE1a stimulates BMP2‐induced osteoblast differentiation IRE1a inhibited BMP2‐induced bone formation through GEP	IRE1α pathway negatively regulate osteogenic differentiation and a regulatory loop of IRE1a, BMP2, GEP, and JunB is involved	^57^
HCSMCs	BMP‐2 activates IRE1α pathway to increase Runx2 expression and XBP1s associates with RUNX2 promoter Inhibition of IRE1α pathway limits BMP‐2‐mediated mineralization of HCSMC	IRE1α‐XBP1s pathway promotes RUNX2 expression to regulate HCSMC calcification	^66^
A7r5, human, rat, and mouse primary VSMCs	XBP1u protein levels are decreased in VSMCs treated with high phosphate, human and mouse arteries with chronic renal failure (CRF) XBP1u inhibits calcification in A7r5, rat primary VSMCs, and human aortic SMCs XBP1u directly binds to β‐catenin and inhibits β‐catenin/ TCF transcriptional activity and the target genes Runx2 and Msx2	XBP1u inhibits vascular calcification by binding to β‐catenin and counteracting β‐catenin‐Runx2/Msx2 signalling	^67^
SM22α‐Cre^+^‐Xbp1^flox/flox^ (Xbp1^SMKO^) mouse	The SMC‐specific XBP1 knockout mice with adenine diet show severe vascular calcification compared to the control group		^67^
Human mesenchymal stem cell line immortalized by expression of the telomerase reverse transcriptase gene (hMSC‐TERT)	IRE1α protein level is upregulated in hMSC–TERT cells after bortezomib and MLN2238 treatment, while silencing of IRE1a abrogates this effect	IRE1α pathway is involved in proteasome inhibitor‐induced osteogenic differentiation	^78^
Mouse bone marrow mesenchymal stem cells (mMSCs), MM patient‐derived mesenchymal stem cells (MM‐MSCs) and MCT3‐E1	Proteasome inhibitor induces osteogenic differentiation and activates IRE1α‐XBP1s signalling XBP1s is involved in proteasome inhibition‐induced osteogenic differentiation and regulates the activation of PERK‐ATF4 ER stress signalling pathway Overexpression of human XBP1s triggers osteogenic differentiation	IRE1α‐XBP1 pathway mediates proteasome inhibition‐induced osteoblast differentiation by direct effects on essential osteogenic genes and positive regulation of another ER stress arm ATF4	^77^
Wild type mouse	Inhibition of IRE1α‐Xbp1 signalling reverses bortezomib‐induced bone formation in mice		^77^
mBM‐MSCs	Inhibition of IRE1α pathway leads to downregulation of bortezomib‐induced p21Cip1 and p27Kip1 during osteogenesis Enforced expression of XBP1s upregulates p21Cip1 and p27Kip1 and induces G0/G1 cell cycle arrest in mBM‐MSCs XBP1s can bind to the promoters of p21Cip1 and p27Kip1	IRE1α‐XBP1s pathway transcriptionally regulate p21Cip1 and p27Kip1, which may be involved in proteasome inhibitor‐induced osteogenic differentiation	^79^
U2OS, (HNOst, HUVEC, The fibroblast‐like synoviocytes (FLS), Dermal fibroblasts)	IRE1α pathway is activated during Hcy‐induced apoptosis Silencing IRE1α expression by siRNA effectively suppresses Hcy‐induced cell death of osteoblastic cells	IRE1α pathway mediates Hcy‐induced cell death	^87^
MG63, hFOBs	The expression or incubation of MEL protein triggered the activation of IRE1α and CHOP	IRE1α pathway mediates MEL‐induced apoptosis of MG63 cells via CHOP signalling	^98^
MC3T3‐E1, human osteoporotic osteoblasts	AGEs activate IRE1α by interfering with GRP78‐IRE1α binding IRE1α knockdown inhibits GA‐induced phosphorylation of JNK and p38, activation of caspase‐3 and ratio of Bcl‐2/Bax compared to control siRNA‐treated cells	IRE1α knockdown improves osteoblast apoptosis by inhibiting IRE1α‐ASK‐JNK axis and p38 phosphorylation	^84^, ^92^

## 4 CHONDROGENESIS

### 4.1 Role of the IRE1α pathway in chondrocyte differentiation

Previous studies have highlighted the role of the IRE1α pathway in osteoblasts,^29^ further identifying that XBP1s contribute positively to regulating endochondral bone formation.^60^ ATDC5 and C3H10T1/2 cell lines were used to explore XBP1 expression profiles during chondrocyte differentiation. Under specific conditions involving high cell density microcapsule culture and exposure to cartilage‐inducible factors, such as growth factor BMP2, these cells undergo differentiation primarily into chondrocytes. During the chondrogenic differentiation process in these cells, XBP1s are activated.^60^ In the ATDC5 cell line and bone marrow stromal cells (BMSCs), BMP activates the core promoter of XBP1s and directly interacts with its promoter region (−407 and +133), thereby working synergistically to promote chondrogenic differentiation.^60^ An ex vivo cell mass transplantation experiment confirms the promotion effect of XBP1s on endochondral bone formation. BMP2 is suggested to activate RUNX2 via the XBP1 pathway, resulting in the osteochondrogenic phenotype observed in vascular smooth muscle and valvular interstitial cells.^98^ These findings indicate that XBP1s are a crucial positive regulator in chondrocyte development processes.

However, other researchers hold opposing viewpoints. One study suggests that IRE1α activation inhibits chondrocyte differentiation, and this effect depends on its enzymatic activity.^30^ The discrepancy could be attributed to different cell types, the experiments of this study were primarily conducted in BMSCs while the above one^60^ focuses on the chondrocyte cell lines. Furthermore, these differences reveal that IRE1α and XBP1s, as two components of IRE1α pathway, may play distinct roles in chondrogenesis, necessitating a comprehensive understanding of the significance of IRE1α pathway in chondrocyte differentiation.

### 4.2 Involvement of the IRE1α pathway in chondrocyte apoptosis

Chondrocyte apoptosis is considered the primary cause of cartilage degeneration and a significant aspect of osteoarthritis (OA) pathology. A positive correlation is observed between the number of chondrocytes experiencing ERS and cartilage degeneration in human OA cartilage. Additionally, the number of caspase‐3‐positive chondrocytes suggests the potential involvement of the IRE1α‐JNK pathway in this context. An IRE1α‐XBP1‐CHOP axis has also been suggested as a pro‐apoptotic pathway in chondrocytes.^99^ Furthermore, IRE1α has been demonstrated to enhance proliferation and inhibit apoptosis in chondrocytes by regulating IκBα phosphorylation and p65 nuclear translocation.^100^

In contrast, the initial protective role of the IRE1α pathway in chondrocytes has been highlighted,^101^ indicating its potential as a protective response during cartilage degeneration. Recent research supports this notion. Moreover, IRE1α pathway activation can suppress ERS‐mediated apoptosis in chondrocytes, chondrosarcoma cells, and OA cartilage. IRE1α dissociates from BiP and inhibits ERS‐mediated apoptosis via p‐JNK, Erk1/2, caspase‐3, and CHOP.^102^, ^103^, ^104^, ^105^ According to a new theory, an IRE1α‐mTOR‐PERK signalling axis is implicated in autophagy inhibition regulation and apoptosis induction.^106^ IRE1α can inhibit mTOR and its downstream target PERK pathway, restoring autophagy and exerting an anti‐apoptosis effect. A study employing a pathological mouse temporomandibular joint OA (TMJ OA) model also demonstrated that the IRE1α pathway functions as a pro‐survival factor.^107^ Thus, the IRE1α pathway plays a dual role in chondrocyte apoptosis. These findings suggest that an optimal activation level of the IRE1α pathway may hold promise for treating cartilage degeneration; however, further studies are warranted to confirm this efficacy.

### 4.3 Role of the IRE1α pathway in cartilage development and pathology

XBP1s may play an important role in chondrocyte hypertrophy, mineralization, and bone length in vivo. Elevated levels of XBP1 expression have been discovered in embryonic and postnatal mouse chondrocytes and growth plates. When exposed to a 5‐day Ad‐XBP1 treatment on a mouse ex vivo model at a 15‐day fetal age, the undifferentiated cartilage explants progressed through all stages of endochondral osteogenesis.^60^ However, within the intricate cartilage environment in vivo, XBP1 is not deemed essential for cartilage hypertrophy. A mouse model, Xbp1^CartDEx2^, was generated by specifically deleting exon 2 of the XBP1 gene in the cartilage.^108^ The Xbp1^CartDEx2^ mouse exhibits cartilage dysplasia and a mild dwarfism phenotype during the early stages of bone development. H&E staining revealed delayed mineralization of the secondary ossification centre in the growth plate and a shortened hypertrophic area. As the Xbp1^CartDEx2^ mouse matures, the observed phenotype disappears. This suggests that XBP1 regulates chondrocyte proliferation, influencing the timing of cartilage maturation and matrix mineralization rather than guiding chondrocyte differentiation during endochondral ossification.^108^ A recent study highlighted IRE1α as a negative regulator influencing chondrocyte proliferation and differentiation in postnatal growth plates. Fan et al. observed that the deficiency of IRE1α leads to excessive chondrocyte hypertrophy by impairing the homeostasis within the Indian Hedgehog (IHH) and parathyroid hormone‐related protein (PTHrP)/parathyroid hormone 1 receptor (PTH1R) feedback loop.^109^

XBP1 signalling activation has also been associated with cartilage pathology in patients with OA and animal OA models.^101^, ^110^ The IRE1/mTORC1/TNF‐α‐regulated inflammatory response in peritoneal macrophages, ERS‐IRE1α‐TXNIP‐NLRP3 axis, and IRE1α‐IκBα‐p65 signalling may represent specific therapeutic targets.^100^, ^111^, ^112^ Contrarily, Kung et al. found that XBP1s‐regulated networks were enriched in the cartilage affected by OA in ColIITg^cog^ (c/c) mice, indicating its early chondroprotective role.^113^ The absence of XBP1s in the mouse model of multiple epiphyseal dysplasias (EDM5) exacerbates the severity of the skeletal phenotype.^114^ This indicates that XBP1 signalling may serve as a therapeutic target for diseases characterized by mutant protein aggregation by regulating autophagy, degradation, and ERAD.^115^ In another study, a ColX^N617K^ (a Schmid metaphyseal chondrodysplasia (MCDS) model)/Xbp1^Cart Δ Ex2^ (C/X) mice was created, exhibiting MCDS phenotype and a specific knockout of XBP1.^116^ When comparing bone parameters in C/X mice to those in ColX^N617K^ mice, quantitative analysis reveals no significant differences in femur length and the length of the hypertrophic zone in pathological expansion. This finding suggests that the IRE1α/XBP1 pathway may not play a significant role in MCDS pathology.^114^ Instead, it may serve an adaptive function in response to ER stress as part of the UPR. Additionally, IRE1α has been reported to regulate chondrocyte inflammation and differentiation via the p38 kinase pathway.^117^ Epstein–Barr virus‐induced gene 3 (EBI3) shows differential expression during chondrogenic differentiation and can be produced by MSC. Increased expression of EBI3 can lead to excessive activation of IRE1α, consequently inhibiting chondrocyte differentiation. This mechanism may contribute partially to the suppression of chondrogenesis observed in patients with RA.^118^ The IRE1α pathway may not be crucial in cartilage development and certain pathological conditions. However, determining the precise role of the IRE1α pathway in vivo in cartilage necessitates further investigation.

**FIGURE 2:**
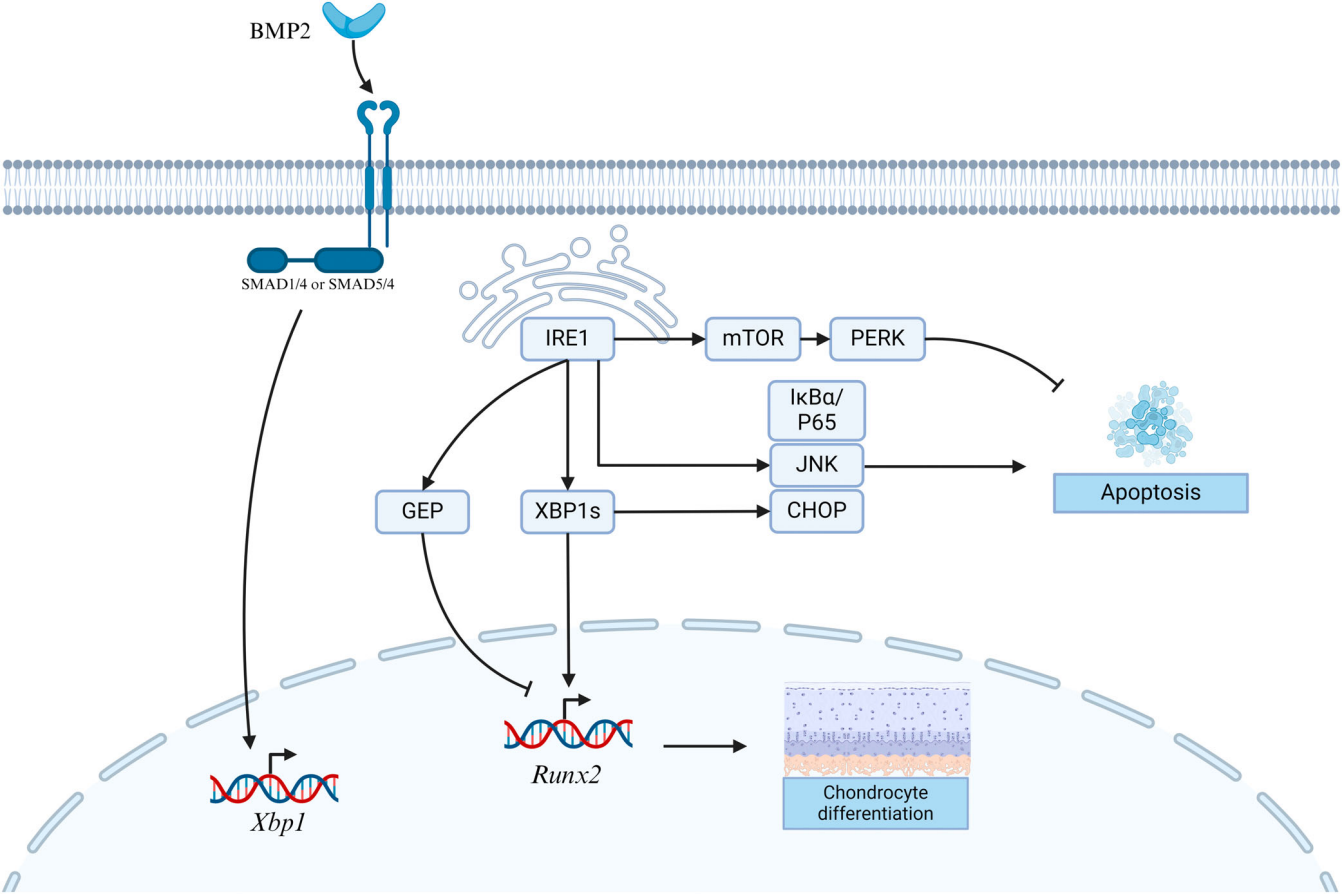
Regulation of the IRE1α pathway in chondrogenesis. Activation of the IRE1α pathway promotes chondrogenic differentiation via XBP1s.

Collectively, the IRE1α pathway play a role in chondrogenesis (Figure 2). Some of the studies involving the IRE1α pathway and chondrogenesis have also been summarized in Table 2.

**TABLE 2:** Role of IRE1α pathway in chondrogenesis.

Cell type/animal model	Results	Conclusion	Reference
BMSCs, C3H10T1/2	IRE1α negatively regulates expression of RUNX2, ColII, Sox9, and ColX, which is dependent on its enzymatic activity IRE1α is induced by GEP and can enhances IHH/PTHrP signalling	IRE1α pathway inhibits the GEP‐mediated chondrocyte differentiation, and the inhibition of RE1α is rely on its enzymatic activities during chondrogenesis	^31^
BMSCs, C3H10T1/2 and ATDC5	XBP1s is upregulated in C3H10T1/2 and ATDC5 during chondrocyte differentiation XBP1S stimulates chondrogenesis in vitro XBP1s is activated by BMP2‐Smads signalling	XBP1s positively regulates chondrocyte differentiation and endochondral bone growth	^62^
Ex vivo models of 15‐day‐old foetal mouse metatarsal bone	High levels of XBP1s are detected in growth plates XBP1s promotes endochondral bone formation ex vivo		^62^
C28I2, C3H10T1/2, ATDC5, and chondrocytes from OA patients	IRE1α pathway is activated during chondrocyte differentiation and OA Overexpressing IRE1α inhibits ER stress‐mediated apoptosis in chondrocyte ATF6 binds to the promoter of XBP1 gene and increases the transactivation of XBP1 gene in chondrocytes induced by TNFα and IL‐1β Knockdown of XBP1s enhances ER stress‐mediated apoptosis in OA cartilage Ad‐XBP1s inhibits apoptosis‐related genes of OA cartilage induced by TNFα	IRE1α‐XBP1s pathway negatively regulates ERS‐induced apoptosis of chondrocytes during chondrocyte differentiation and OA	^105^, ^106^
Primary human knee chondrocytes	Melatonin inhibits ERS–Related human primary chondrocyte apoptosis by inhibiting the IRE1α‐XBP1S‐CHOP signalling pathway in chondrocytes	IRE1α‐XBP1S‐CHOP signalling is responsible for chondrocyte apoptosis in OA	^102^
Rat primary chondrocytes	IRE1α Inhibits mTOR, promotes autophagy, and reduces ER stress‐apoptosis mTOR inhibits autophagic flux and activates PERK to induce ER Stress‐apoptosis	IRE1α‐mTOR‐PERK axis is involved in the regulation of autophagy and apoptosis in chondrocytes	^109^
Rat primary chondrocytes	Salicin (SA) inhibits IRE1α‐IκBα‐p65 signalling‐mediated ER stress by directly binding to IRE1α	SA inhibited IRE1α‐mediated ER stress and subsequently promoted chondrocyte proliferation, decreases inflammatory factor expression, and inhibits chondrocyte apoptosis	^103^
ACTL‐induced OA model	SA intra‐articular injection ameliorates ACLT‐induced OA progression by inhibiting IRE1α‐mediated ER stress		^103^
Human OA cartilage samples	XBP1 splicing and GRP78 expression in severe OA chondrocytes were similar to the levels in mild OA, however, XBP1 splicing was higher in moderate OA than in mild and severe OA	IRE1α‐ XBP1s pathway may play a preliminary protective role in mild OA cartilage, which is reduced in severe OA	^104^
Human primary chondrocytes	Tunicamycin dose‐dependently increased CHOP expression and apoptosis of chondrocytes. Although tunicamycin upregulated XBP1 splicing in chondrocytes, its impact on XBP1 splicing was weakened at higher concentrations		^104^
Xbp1^flox/flox^. Col2a1‐Cre (Xbp1^CartDEx2^) mouse	Xbp1CartDEx2 mice exhibit mild dwarfism and delayed ossification Chondrocyte proliferation is dysregulated in the Xbp1CartDEx2 growth plate but cell death is not	XBP1 regulates chondrocyte proliferation and the timing of cartilage maturation and matrix mineralization during endochondral ossification	^111^
Mouse primary chondrocytes	IRE1α is hyperactivated in the Xbp1CartDEx2 growth plate but does not cause RIDD		^111^
ColX^N617K^‐ Xbp1^CartΔEx2^ mouse	Neither dwarfism nor the hypertrophic zone expansion of ColXN617K is significantly altered by loss of XBP1 activity in C/X chondrocytes ER stress‐induced apoptosis and developmental arrest of ColXN617K chondrocytes are regulated independently of XBP1	XBP1 signalling has no effect on the overall severity of the disease phenotype of MCDS	^119^
Xbp1^Col2CreΔex2^‐Matn3^V194D^ mouse	The loss of XBP1 from cartilage leads to an exacerbation of the EDM5 phenotype Ablation of XBP1 affects cartilage growth plate morphology and chondrocyte apoptosis XBP1 modulates the aggregation of mutant matrilin‐3 in the chondrocytes of Matn3^V194D^ mice	XBP1 signalling plays an important role in modulating levels of protein aggregation and EDM5 pathology	^118^

## 5 OSTEOCLASTOGENESIS

### 5.1 IRE1α pathway directly promotes osteoclast differentiation

The osteoclast, pivotal in bone metabolism, represents a type of cell responsible for bone resorption and reconstruction. It originates from mononuclear/macrophage cells.^119^, ^120^ During osteoclastogenesis, numerous active proteins are synthesized, inducing ER stress and UPR.^121^ Studies indicate that the ER stress activation by thapsigargin, an ER stress inducer, can promote osteoclastogenesis through the IRE1α pathway.^122^, ^123^ Monocyte chemotactic protein‐1 induced protein (MCPIP), categorized as a kind of zinc finger protein, is induced by monocyte chemotactic protein‐1. IRE1α has been found to play a role in MCPIP‐induced osteoclast precursor differentiation. MCPIP‐induced reactive oxygen species (ROS) triggers the induction of ER stress and activation of IRE1α. Conversely, inhibiting IRE1α suppresses TRAP, CTSK, and autophagy marker expression, leading to impaired osteoclastogenesis.^124^, ^125^

The nuclear factor of activated T cells cytoplasmic 1 (NFATc1) is one of the essential transcription factors for osteoclast development.^126^ Its promoter region contains two UPR element‐like sequences (5′‐GAAG‐3′).^127^ A recent study highlights the significant role of the IRE1α/XBP1 pathway in osteoclast formation by regulating the activation of NFATc1. During the early stages of osteoclast differentiation, the activation of the IRE1α‐XBP1 pathway reaches its peak. XBP1s exhibit the capacity to bind to the NFATc1 promoter directly in bone marrow‐derived macrophages (BMMs) and RAW264.7 cells, promoting its expression.^31^ Moreover, a recent study revealed that MM‐extracellular vesicles promote osteoclastogenesis through the IRE1α‐XBP1‐NFATc1 axis.^128^ Tohmonda et al. created Ern1^flox/flox^/Mx1^Cre^ mice (Ern1^Mx1^) utilizing Cre recombinase under the regulation of the IFN‐inducible Mx1 promoter.^129^ The long bone parameters (BV/TV, Tb.Th, and Tb.N) in Ern1^MX1^ mice increased more than in WT mice. This is linked to decreased osteoclast activity of bone marrow macrophages caused by IRE1α deficiency.^31^

Like osteogenic differentiation, an excess of protein synthesis might partially contribute to activating the IRE1α pathway during the osteoclast differentiation. However, another mechanism elucidates the induction of ER stress and the IRE1α pathway. Previous studies demonstrated that RANKL signalling can trigger Ca^2+^ influx into the ER via Inositol 1,4,5‐trisphosphate receptor type 2 and 3 (ITPR2 and ITPR3), thus promoting NFATc1 activation.^126^, ^130^ Fluctuations in Ca^2+^ within the ER can potentially cause ERS.^131^ During the osteoclast differentiation process, IRE1α/XBP1 pathway activation has been confirmed to partially depend on the Ca^2+^ oscillations mediated by ITPR2 and ITPR3.^31^ In summary, an ITPR2/3‐IRE1‐XBP1s‐NFATc1 axis plays a significant role in osteoclast differentiation. Moreover, the upstream mechanism that transfers osteoclastogenic stimulation to the IRE1α pathway has been partially elucidated.^57^ Cytohesin‐2 functions as a guanine nucleotide exchange factor that activates ADP ribosylation factor 1 and 6 (ARF1 and ARF6).^132^ Inhibiting the cytohesin‐2/ARF1 axis using SecinH3 or silencing cytohesin‐2 has been observed to inhibit IRE1α activity, consequently enhancing osteoclast formation. Unlike the downstream role of JNK in the IRE1α pathway, JNK has been revealed to function as an upstream activator of IRE1α, regulating XBP1 expression. During the osteoclast differentiation, both JNK and IRE1α pathways are inhibited by SecinH3.^57^ While the precise mechanism underlying how the JNK pathway regulates IRE1α endonuclease activity remains unknown, a cytohesin‐2/ARF1/JNK/IRE1α axis in osteoclast differentiation is established. This axis may serve as a supplementary mechanism upstream of the IRE1α/NFATc1 pathway mentioned earlier. The IRE1α pathway, as a crucial component of osteoclastogenesis, holds potential as a therapeutic target for conditions such as osteoporosis, myeloma, and tumours exhibiting bone metastasis.

### 5.2 IRE1α pathway regulates osteoclast differentiation indirectly

RANKL, expressed by osteoblasts, hypertrophying chondrocytes, activated T lymphocytes, and synovial fibroblasts (SFs),^133^ serves as key regulators in osteoclast formation.^134^ RANKL binds to the receptor activator of nuclear factor kappa B in osteoclast progenitors, promoting osteoclast differentiation and activity. Recent studies have revealed that the IRE1α pathway promotes osteoclast differentiation indirectly by upregulating RANKL expression in osteoblasts. The PTH1R—a member of the G protein‐coupled receptor family—serves as a shared receptor for parathyroid hormone (PTH) and PTHrP.^135^, ^136^ Their activation promotes osteoclast development and bone resorption by elevating RANKL expression in osteoblasts and stromal cell.^137^ A recent study found that in osteoclasts, IRE1α/XBP1s positively regulate PTH1R transcription by directly binding to the P2 promoter of PTH1R. This enhances cell responsiveness to PTH, consequently raising RANKL expression to promote osteoclast development.^138^

The primary causes underlying joint replacement failure and the need for revision surgery often stem from osteolysis induced by wear particles, leading to subsequent aseptic loosening. Fibroblasts are the most important cell type on the periprosthetic membrane, playing a crucial role in responding directly to debris. They initiate the production of proinflammatory cytokines and RANKL, thereby contributing to osteoclast formation.^139^ Furthermore, recent studies have revealed the involvement of the IRE1α pathway in the pro‐osteoclast effect exhibited by fibroblast. When stimulated by TiAl6V4 particle (TIPS), fibroblasts exhibited significantly elevated IRE1α and CHOP expression, increased RANKL levels, and augmented ratio of RANKL/osteoprotegerin (OPG). However, the inhibition of ERS reversed RANKL upregulation.^140^ In TIP‐treated mice, XBP1s were also implicated in the degenerative process associated with TIP‐induced osteolysis.^141^ Recent research has demonstrated the activation of IRE1α in periosteal tissue from clinical specimens in four patients with osteolysis.^142^ In patients with aseptic loosening, a significant increase was observed in XBP1 expression levels on the periprosthetic membrane. This elevation was also found in SFs activated by TIPs. Both siRNA‐XBP1s and the IRE1α endonuclease inhibitors 3‐E‐5, 6‐D exhibit the ability to suppress TIP‐induced osteoclast differentiation in vitro and in vivo through reducing RANKL expression and RANKL/OPG ratio in SFs. Under the MM microenvironment, the XBP1 level is elevated, leading to increased RANKL expression in BMSCs.^143^ Conversely, inhibiting XBP1s results in impaired osteoclast differentiation. The activation of the IRE1α pathway in BMSCs within the MM microenvironment is significant. This activation enhances the expression of RANKL, VCAM‐1, and IL‐6, promoting osteoclast formation and MM cell growth.^143^ While the precise mechanism underlying how XBP1s influence the expression of RANKL requires further elucidation, targeting the blockade of the IRE1α‐XBP1 pathway may be a potential therapy strategy for wear particles‐induced osteolysis and MM.

**FIGURE 3:**
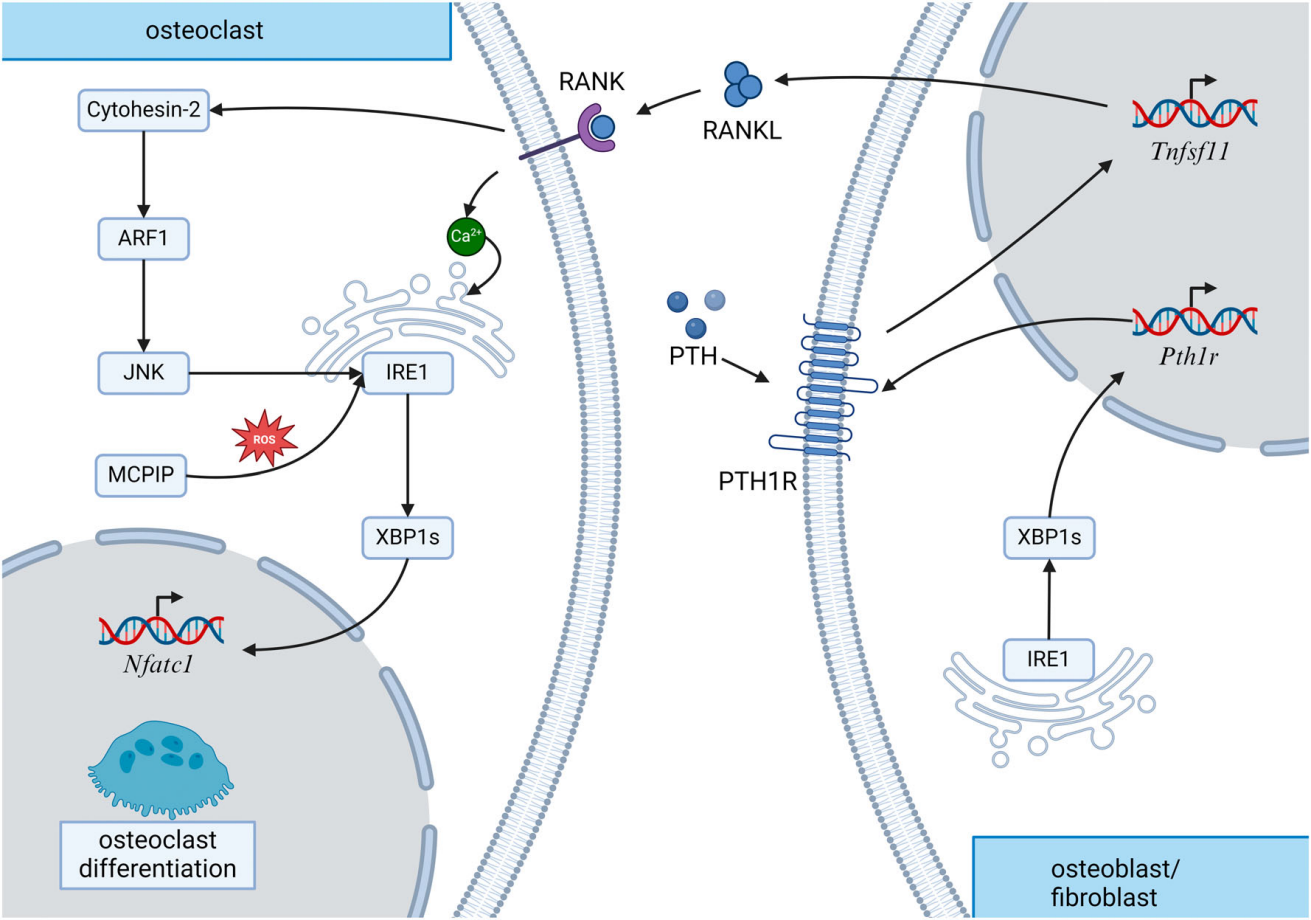
IRE1α pathway regulation in osteoclastogenesis. The IRE1α pathway regulates osteoclastogenesis through direct and indirect mechanisms.

All the IRE1α pathway induced molecular mechanisms involved in osteoclastogensis have been schematically shown below (Figure 3) and some of the research involving the IRE1α pathway in osteoclastogenesis has been summarized in Table 3.

**TABLE 3:** Role of IRE1α pathway in osteoclastogenesis.

Cell type/animal model	Results	Conclusion	Reference
Mouse primary BMMs	IRE1α‐XBP1s pathway is transiently activated during osteoclast differentiation Knockdown or knockout of IRE1α‐XBP1s signalling leads to impaired osteoclast differentiation XBP1s binds to the promoter of the Nfatc1 gene and promotes Nfatc1 transcription The activation of IRE1α during osteoclastogenesis is dependent on Ca2+ influx mediated by ITPR2 and ITPR3	IRE1α‐XBP1 pathway is a critical regulator to promote osteoclast differentiation by enhancing Nfatc1 transcription	^32^
Ern1^flox/flox^/Mx1Cre (Ern1^Mx1^) mouse	The Ern1Mx1 mouse shows higher bone mass compared to control group The mice reconstituted with BM cells isolated from Ern1Mx1 mice had higher bone mass compared with those reconstituted with WT BM cells		^32^
RAW264.7 and human primary osteoclasts	IRE1α‐XBP1 pathway is activated in Raw264.7 cells treated with MM‐EVs The chemical block of the IRE1α‐XBP1 pathway perturbs MM‐EV‐induced osteoclast differentiation	IRE1α‐XBP1 pathway promotes osteoclast MM‐EVs‐induced differentiation	^131^
Mouse primary BMMs	Cytohesin‐2 inhibition reduces the endoribonuclease activity of IRE1α and osteoclast differentiation The JNK pathway acts upstream to control the endoribonuclease activity of IRE1	The cytohesin‐2/ARF1/JNK/p‐IRE1 pathway play an important role in osteoclast differentiation	^58^
MEFs and mouse primary osteoblasts (POBs)	Pth1r expression is suppressed in IRE1α^−/−^ mEFs XBP1s binds to the P2 promoter of Pth1r and induces transcriptional activity Silencing of Xbp1 suppresses PTH‐induced RANKL expression and osteoclastogenesis	IRE1α‐XBP1 pathway is essential for PTH‐induced Rankl expression in primary osteoblasts and thereby promotes osteoclast formation	^141^
Synovial fibroblasts (SF)	IRE1α pathway is activated by TiPs in human synovial fibroblasts XBP1s mediated the upregulation of RANKL induced by TiPs and promoted osteoclastogenesis in SFs	XBP1s induces RANKL expression in fibroblasts	^143^, ^144^
Wear particle‐induced calvarial osteolysis mice model	Upregulation of XBP1s is detected in peri‐implant membrane and TiPs‐treated animals XBP1s mediated the upregulation of RANKL and osteoclastogenesis and inhibition of IRE1α reverse it in animal models		^143^, ^144^
Human kidney 297T cells and human BM stromal cell lines KM101 cells	XBP1s are induced in BMSCs of the MM microenvironment XBP1s overexpression in BMSCs promotes BMSC support of osteoclast formation Xbp1 knockdown reduces BMSC secretion of RANKL and supports of osteoclast formation	XBP1s is a pathogenic factor underlying BMSC support of osteoclast formation through promoting expression of VCAM‐1, IL‐6, and RANKL	^146^

## 6 IRE1α IN BONE IMMUNE CELLS

### 6.1 Macrophage

As one of the most crucial parts of the innate immune system, macrophages also play a role in tissue repair, homeostasis, and development.^144^ Macrophage is considered to be a precursor of osteoclast,^119^ as well as a crucial regulator in bone inflammation and metabolism.

Type I interferon (IFN‐α/β) are now recognized to have diverse immunoregulatory effects activating macrophages and natural killer cells, promoting T cell survival and dendritic cell (DC) maturation, and increasing the production of Th1‐polarizing cytokines to mediate innate and adaptive immune responses.^145^ IFN‐β functions as a negative regulator of osteoclast differentiation and plays a crucial role in maintaining osteo‐immune homeostasis even at the constitutive level.^146^ In primary macrophages, macrophage‐like cell lines (RAW264.7), fibroblasts, and HEK293 cells, overexpression of XBP‐1s coupling with ER stress enhances LPS‐induced IFN‐β, while the absence of XBP‐1 completely prevented synergistic induction of IFN‐β.^147^ These findings suggest that XBP1s play a crucial role in UPR synergistical enhancement of IFN‐β induction in these cells. Zeng et al. further elucidated this mechanism that XBP1 binds a potential enhancer element 6 kb distal to the ifnb1 gene to enhance its promoter activity during concomitant ER stress and TLR4 stimulation,^148^ indicating XBP1s in macrophage may plays a negative role during bone absorption through regulating IFN‐β.

On the contrary, IRE1α pathway is also proven to facilitate inflammatory cytokines production of macrophages which promotes bone destruction. XBP1 was also found as a positive regulator of innate immunity to tame *Pseudomonas aeruginosa* challenges by enhancing autophagy and bacterial clearance, which facilitated NF‐κB activation to elicit the release of pro‐inflammatory cytokines predominantly in macrophages.^149^ Recognition of pathogen‐associated molecular patterns by the host organism occurs through a group of receptors referred to as pathogen recognition receptors, the best‐studied of which are the Toll‐like receptors (TLRs). According to Martinon et al., TLR2 and TLR4 triggered the IRE1α pathway, which was necessary for optimal and sustained production of pro‐inflammatory cytokines in macrophages.^150^ Macrophage is also a key source of inflammatory cytokines in rheumatoid arthritis (RA), in which IRE1α pathway is involved.^32^ TLR‐induced IRE1α by mediating TRAF6 in macrophages and neutrophils, whereas lacking of IRE1α leads to impaired TLR‐induced cytokine production. Besides, IRE1α can drive metabolic reprogramming in infected macrophages^151^ and promote expression of HIF‐α, IL‐23, and IL‐1β.^152^, ^153^, ^154^ IL‐1β and TNF‐α are important pro‐inflammatory cytokines, which can also contribute to bone loss. It has been discovered that IRE1α pathway activated by ER stress promotes IL‐1β and TNF‐α expression in macrophages. This upregulation of IL‐1β and TNF‐α induced by IRE1α is mediated by GSK3b and XBP1s separately.^155^ This kind of effect of promoting proinflammatory cytokines is also observed in alveolar macrophages.^156^ The IRE1‐XBP1 pathway causes an elevated metabolic state and increased M1 macrophage production of TNF and IL‐6 in cystic fibrosis.^157^ Conversely, inhibition of the ER stress‐associated IRE1α/XBP1 signalling pathway suppresses M1 polarization and ameliorates LPS‐induced lung injury.^158^, ^159^ These findings demonstrate that the IRE1α pathway is a key regulator of macrophage production of pro‐inflammatory cytokines, suggesting a possible function in osteo‐immune homeostasis and the pathophysiology of bone diseases.

### 6.2 T cell

T cells, as a crucial part of adaptive immune system, have been reported to play a role in bone metabolism. CD8(+) cytotoxic T cells directly lyse infected or mutated cells. CD4(+) T helper (Th) regulates other immune cells such through surface receptors and secreted cytokines. On the one hand, activated T cell promotes osteoclastogenesis through expressing RANKL,^160^, ^161^ on the other hand, T cell induce BMSC to differentiate into osteoblast to accelerate bone turnover.^162^ Th17 was also reported to secret IL‐17, which induces osteogenic differentiation of MSCs and RANKL expression of osteoblasts,^163^, ^164^, ^165^ indicating that T cells regulate bone metabolism through releasing cytokines.

IRE1α was detected active in CD4(+) CD8(+) double‐positive thymic T cells and CD8(+) cytotoxic T‐cell population,^166^ then XBP1s was proved to be beneficial for the differentiation of end‐stage CD8(+) T cell.^167^ XBP1s was also reported to be involved in cholesterol‐induced CD8(+) T cell exhaustion,^168^ which represents as a promising therapeutical target to tumour like MM. Besides, XBP1 in DC is considered to promote the activation of CD8(+) T cell in therapeutic settings.^169^

Kemp et al. found that IRE1α was activated during CD4(+) T cell activation and contributed to the production of IL‐4.^33^ According to a recent study, 4μ8c treatment inhibited IL‐5 production when applied to differentiated human Th2 cells and a mouse Th2 cell line.^170^ Another study demonstrated that inhibition of IRE1α kinase activity can maintain Treg stability and function,^171^ indicating a negative role of IRE1α‐p38 in Treg stability. Similarly, IRE1α plays a role in the ER stress‐induced CD4(+) T cell apoptosis in Septic mice through a mTOR–Akt‐IRE1α‐JNK signalling.^172^ Besides, XBP1 was indispensable in the Th2 lineage commitment for its involvement in Th2 polarization,^173^, ^174^ considering Th2 as source of IL‐4 and IL‐13 which show significant role in arthritis,^175^ the immune effect of IRE1α pathway in T cells can also be a therapeutical target for arthritis treatment and prevention.

### 6.3 B cell

B cells are also part of the adaptive immune system, which can be activated into antibody‐secreting plasma cell. Their primary function is to produce antibodies to fight pathogens and present antigens for T‐cell activation and secrete various cytokines. Activated B cells can secret RANKL under inflammatory conditions, thereby activating osteoclast formation.^176^ In contrast, under physiological conditions, cells of the B lineage were found to be responsible for 64% of total BM osteoprotegerin (OPG) production, which inhibits osteoclast differentiation.^177^, ^178^ B‐cell lymphoma 3 (Bcl‐3) is regarded as an environment‐dependent cell response regulator that has dual roles in the development of B cells^179^ and also inhibits BMSCs senescence, promoted osteogenesis, and decreased adipogenesis.^180^ It was demonstrated that B cells inhibit osteoblast differentiation in RA.^181^ Moreover, a recent review introduced the role of B cells in periodontitis.^182^

Hu et al. found that B cells fail to signal effectively through the B cell receptor without XBP‐1.^183^ Deletion of XBP‐1 exerts no influence on the number, proliferation, and function of B cell, but XBP1 is recognized indispensable for the terminal differentiation of B lymphocytes to plasma cells.^34^ The role of RIDD of IRE1α pathway in regulating synthesis and secretion of immunoglobulin and antibodies has also been verified.^184^, ^185^ The research evidence presented above suggests that IRE1α may serve as a potential link between immune cells and bone metabolism, despite the paucity of studies on the effects of the IRE1α pathway on B cells and the osteo‐immune microenvironment.

## 7 IRE1α PATHWAY‐RELATED BONE DISEASES

### 7.1 Osteoarthritis

Osteoarthritis (OA), the most common joint disease, is characterized by joint degeneration, which is a process that includes progressive loss of articular cartilage followed by attempted repair of articular cartilage, remodelling and sclerosis of subchondral bone, and osteophyte formation.^186^

ER stress is demonstrated to be involved in the pathology of OA, XBP1 mRNA splicing was increased in moderate human OA cartilage but not in mild or severe cartilage.^101^ It was found that absent of IRE1α or XBP1 in chondrocytes spontaneously resulted in OA‐like cartilage destruction and accelerated OA progression in a surgically induced arthritis model,^187^, ^188^ indicating its initial protective role in OA chondrocytes. Guo et al. confirm the protective role of XBP1s, which was proved to be a negative regulator of apoptosis‐related signalling like caspase cascade and CHOP in TNF‐α‐induced C‐28/I2 cells and OA cartilage transplants.^103^ Liang et al. further revealed that XBP1s protect articular cartilage through TNF‐α/ERK1/2 signalling and further maintains collagen homeostasis by regulating type II collagen expression.^188^ On the other hand, protective role of IRE1α in OA chondrocytes has been further explained because another study found that it suppresses the mechanistic target of rapamycin complex 1 (MTORC1) by the AMPK pathway, promoting autophagy in ATDC5 cell.^107^ Moderate‐intensity exercise can help delay the development of OA. At the early time points of exercise, IRE1α was activated to inhibit mTOR and then promote autophagy and delay the apoptosis of OA chondrocytes.^106^ It was reported that the RIDD of IRE1α pathway inhibits IL‐4 and IFN‐γ production in iNKT cells by promoting t‐bet and gata‐3 mRNA degradation, thereby ameliorating iNKT‐mediated arthritis phenotype.^189^

Additional to the protective role, IRE1α pathway was also proved to contribute to the inflammation and apoptosis in OA.^99^, ^100^ Qin et al. demonstrated that melatonin promotes SIRT1 expression and inhibits ERS and apoptosis of OA chondrocytes by blocking the IRE1α‐XBP1S‐CHOP signalling pathway.^99^ Zhu et al. found that Salicin (SA) alleviates OA by directly binding to the ER stress regulator IRE1α and inhibits IRE1α‐mediated ER stress via IRE1α‐IκBα‐p65 signalling in chondrocytes.^100^

Above all, IRE1α pathway has two distinct functions in OA. Moderate level of activated IRE1α protect chondrocyte while excessive activation may lead to apoptosis. Therefore, grasping the optimal extent of its activation may be the key to treatment for OA targeting IRE1α.

### 7.2 Rheumatoid arthritis

RA is a prototypic autoimmune joint disease, it is widely accepted that joint inflammation and damage result from the interplay and activation of the resident SF by self‐reactive immune cells.^190^ XBP1 is activated in the SFs of patients with active RA in a TLR‐dependent manner. RA SF can respond to TNF secretion by inducing XBP1s, and XBP1s promote inflammatory cytokine secretion to consequently form an autocrine loop.^191^ Inhibition of XBP1 by miR‐34a‐5p inhibit the secretion of TNF‐α and IL‐6 by FLS but also inhibits the proliferation of fibroblast‐like synoviocytes (FLS).^192^ Furthermore, through controlling autophagy, downregulation of IRE1α promotes the growth and invasion of TNF‐α‐induced RASFs.^193^ The E3 ubiquitin ligase synoviolin (SYVN1) functions as an anti‐apoptotic factor that is responsible for the outgrowth of synovial cells during the development of RA.^194^ Collagen‐induced arthritis (CIA), similar to RA, is characterized by overgrowth of articular synovial cells. Transient expression of IRE1α significantly enhanced apoptosis of SFs from both normal and CIA mice, and elevated SYVN1 expression facilitates the hyperproliferation of synovial tissues by promoting ubiquitination and degradation of IRE1α during the development of arthritis.^195^ Thus, blocking IRE1α could be a therapeutic approach to prevent SF from overgrowth.

In RA, macrophage is one of the major sources of inflammatory mediators. XBP1s mRNA was found to be higher in RA patients' synovial fluids than in control.^32^ It was also detected that the expression of GRP78, IRE1, and XBP1s were increased in PBMCs of RA patients compared with healthy controls.^196^ Conditional deletion of IRE1α in myeloid cells leads to inhibition of inflammation in arthritis. When BMMs from conditional IRE1α knockout mice were stimulated with TLR agonists, the production of inflammatory cytokines, including IL‐1b, IL‐6, TNF‐α RANTES, and ISG15, was significantly reduced compared with control.^32^ According to a recent research, targeting IRE1α can ameliorate adjuvant‐induced arthritis through IRE1/mTORC1/TNF‐α‐regulated inflammatory response initiated in peritoneal macrophages.^111^ This indicates that IRE1α pathway might regulates inflammatory cytokines of macrophage in RA. Researchers further show that the RIDD targets (miRNA‐17, ‐34a, ‐96, and ‐125b) were downregulated in RA samples compared with healthy control.^196^ The RNase activity of IRE1α may present a new opportunity to improve existing therapeutic and/or diagnostic markers in RA patients, even though the role of RIDD in RA needs further clarification.

### 7.3 Spinal related diseases

Spondyloarthropathies (SpA) are a cluster of interrelated and overlapping chronic inflammatory rheumatic diseases that primarily include AS, reactive arthritis, psoriatic arthritis, and inflammatory bowel disease arthritis.^197^ Expressions of GRP78, CHOP, and XBP1 in monocytes/macrophages of SpA peripheral blood and synovial fluid were higher than those in healthy controls and patients with OA.^198^ AS is a type of chronic arthritis characterized by inflammatory spondylitis, peripheral arthritis, and enthesitis. Syndesmophyte formation is part of AS which may leads to disability.^199^ Tissue‐nonspecific alkaline phosphatase (TNAP) is responsible for the abnormal mineralization of AS MSCs,^66^ the authors identified retinoic acid receptor‐β (RARB) as a transcription factor with the potential to regulate TNAP expression. While application of 2 independent XBP1‐shRNAs can abolish abnormal mineralization. The authors further reveal that XBP1 binds to promoter of RARB to regulate TNAP expression, establishing a XBP1s/RARB/TNAP axis in AS syndesmophyte pathogenesis.^66^

Intervertebral disc degeneration (IDD) is a degenerative status of spine with ageing, which mainly starts from nucleus pulposus. The pathological mechanism of IDD is mainly caused by the destruction of metabolic homeostasis in the nucleus pulposus. Loss of nucleus pulposus cells (NPCs) and decrease of protein synthesis could result in the degradation of ECM and break the homeostasis in intervertebral disc (IVD).^200^ Once the steady state in metabolism is lost, imbalance in anabolic and catabolic events often goes along with an inflammatory reaction, which leads to further matrix breakdown.^201^ IL‐1β and TNF‐α, which were found highly expressed in NPCs in IDD patients, were used to treat NPCs, it was detected that IRE1α and PERK pathway are activated in degenerated‐NPC. The synthesis proteins of Agg, Col2, and Sox9 were significantly rescued in IRE1‐α siRNA‐transfected D‐NPCs compared with the controlled group caused by IL‐1β and TNF‐α treatment.^202^ Kang et al.^203^ further explain the involvement of IRE1α in IDD. Inhibition of IRE1α by 4μ8c or siRNA suppresses IL‐1β‐induced matrix degeneration and ECM degeneration of NP cells, as well as ameliorating IVD degeneration in vivo. It is reported that inhibition of IRE1α suppressed the degeneration of NP cells in vitro and in vivo through regulating IL‐1β‐induced ROS level, NF‐κB, PI3K/Akt, and MAPK pathways,^203^ indicating IRE1α may be a potential target for IDD treatment.

## 8 CONCLUSION AND PROSPECTS

This review underscores the pivotal role of the IRE1α pathway in maintaining bone metabolism. IREI α is implicated in osteoblast, osteoclast, and chondrocyte development. The major downstream mechanism of the IRE1α pathway involved in osteogenesis, chondrogenesis, and osteoclastogenesis were delineated, among which XBP1, JNK, and RIDD play a role in regulating the differentiation, viability, and metabolism of bone and immune cells (Figures 1, 2, 3). Osx, RUNX2, NFATc1, RANKL, and PTHR exhibit varying degrees of crosstalk with the IRE1α pathway, contributing to maintaining normal bone metabolism. The IRE1α pathway has shown the potential to enhance osteogenesis, chondrogenesis, and osteoclastogenesis. However, most studies investigating this system rely on evidence from in vitro experiments, with limited availability of in vivo research. Additional in vivo research is crucial to confirm the precise function of IRE1α in the skeletal system. This is imperative as cellular signalling transduction in a complex in vivo environment may exhibit substantial differences compared to that observed in vitro.

In addition, we also present an overview of the role of IRE1α in osteo‐ immunology and its role in some bone diseases. IRE1α pathway can regulate the development and function of macrophages, T cells, and B cells. It can regulate the development of inflammatory arthritis by mediating the inflammation caused by macrophages.^32^ However, due to the lack of direct studies, whether IRE1α pathway influences bone metabolism by regulating other immune cells needs to be further explored. The role of IRE1α in OA, RA, and spinal related diseases has also been widely explored, suggesting that it can be a new therapeutic target combined with novel therapy like organoid extracellular vesicles and bone‐targeted exosomes to treat bone‐related diseases.^204^

Since there have been few drug trials targeting the IRE1α pathway in bone metabolism, its clinical value and side effects have not been well explored. In order to better develop drugs targeting IRE1α and its downstream, more in vivo and functional experiments under diverse pathological conditions are needed. Fortunately, several compounds (bortezomib, melatonin, salicin, etc.) have been found to regulate the IRE1α pathway, directly or indirectly contributing to the attenuation of bone‐related diseases. However, the safety and effectiveness of these drugs have not been guaranteed by clinical trials. The application of the therapy targeting the IRE1α pathway has a long way to go.

The IRE1α pathway, a component of the UPR pathway, is garnering growing attention for its role in bone metabolism. However, future research should explore the precise mechanism of the IRE1α pathway in osteoblast, chondrocyte, and osteoclast. Moreover, osteo‐immunology also represents a promising research direction for investigating the relationship between IRE1α and bone metabolism. With increasing awareness of bone‐related disorders, exploring the IRE1α pathway for treatment is poised to become a research hotspot. The potential of utilizing the IRE1α pathway in treating bone‐related disorders and exploring its potential mechanisms will require comprehensive evaluation. The goal is to develop novel approaches and clinical treatment strategies. In conclusion, numerous prospective avenues exist for research on the IRE1α pathway and its association with bone metabolism. Nonetheless, future studies are necessary to investigate its utility value and underlying mechanism, thereby continuing to contribute to human bone health.

## 9 METHOD

We searched IRE1α pathway‐related literature through utilizing the well‐established databases of Pubmed, Scopus, and Web of Science according to the following keywords related to bone metabolism (osteoblast, chondrocyte, osteoclast, and bone).

## AUTHOR CONTRIBUTIONS

*Conceptualization*: Chengbo Yu and Ke Song. *Writing‐Original Draft Preparation*: Chengbo Yu and Ke Song. *Writing—Review and Editing*: Ke Song, Yong Liu, and Ollie Yiru Yu. *Methodology*: Zhixiang Zhang and Li Xiao. *Visualization*: Lianyi Xu, Zhixing Zhang, and Mi Ai. *Supervision*: Ke Song, Yong Liu, Yingguang Cao and Ying Qing. *English language editing*: Ollie Yiru Yu. *Funding Acquisition*: Ke Song, Zhixing Zhang, and Ying Qing.

## FUNDING INFORMATION

This work was supported by grants from National Natural Science Foundation of China (No. 82170933), Natural Science Foundation of Hubei Province for Distinguished Young Scholars (No. 2023AFA106), Natural Science Foundation of Hubei Province (No. 2022CFB293) and the foundation of affiliated Tongji Hospital, Tongji Medical College, Huazhong University of Science and Technology (No. 2022B22).

## CONFLICT OF INTEREST STATEMENT

The authors declare no conflict of interest.

## Data Availability

The data that support the findings of this study are available from the corresponding author upon reasonable request.
